# Supplementary material for: A class of extracellular vesicles from breast cancer cells activates VEGF receptors and tumour angiogenesis
Source: Nat Commun. 2017 Feb 16;8:14450. doi: 10.1038/ncomms14450 (PMC5316898; doi:10.1038/ncomms14450)
Supplement: Supplementary Information — Supplementary Figures. [file ncomms14450-s1.pdf]

A.

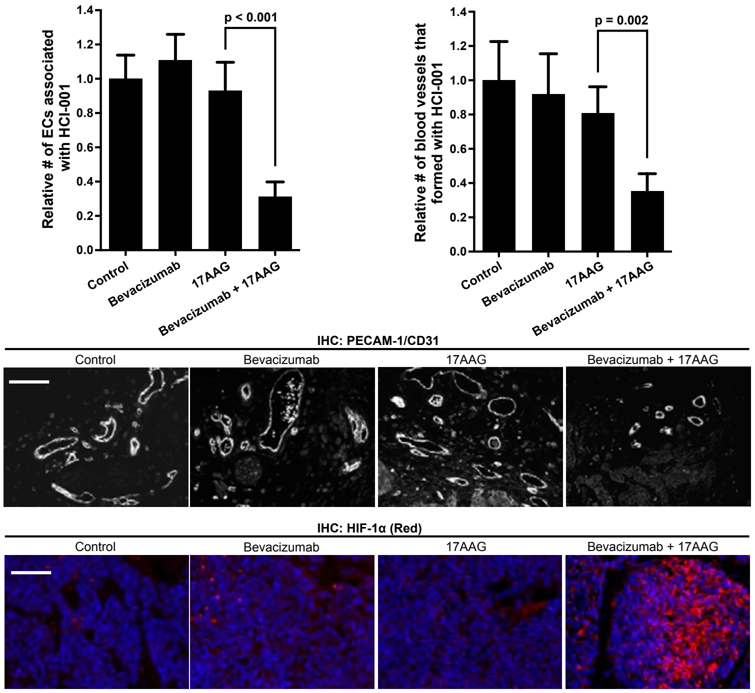

B.

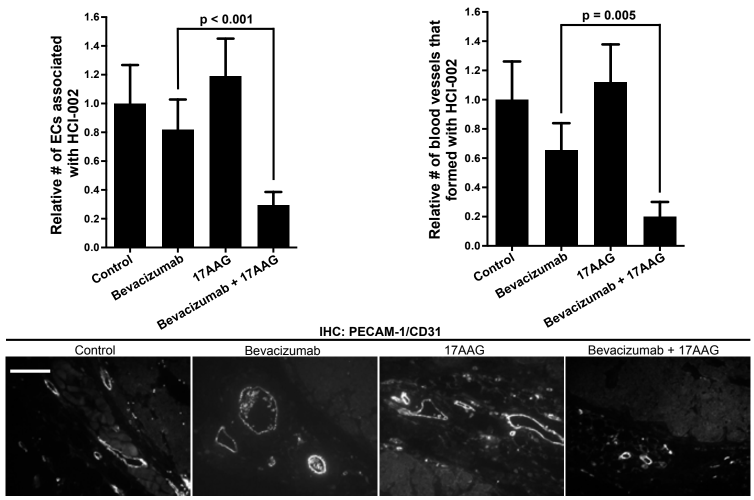

Supplementary Figure 1. A, Top: Histograms showing the effects of co-administering Bevacizumab and 17AAG on blood vessel formation in HCI-001 tumor grafts. The resulting HCI-001 tumors that formed in the experiment shown in Figure 1B, 8 weeks after implantation, were fixed, sectioned, and stained with a PECAM-1/CD31 antibody to detect endothelial cell/blood vessels. The relative endothelial cell counts and blood vessel numbers for sample HCI-001 were plotted. Middle: Images of blood vessel staining for each condition. Scale bar: 100 μm. Bottom: Images of HIF-1a/DAPI staining (for hypoxia) for each condition. Scale bar: 50 μm. **B,** Top: Histograms showing the effects of co-administering Bevacizumab and 17AAG on blood vessel formation in HCI-002 tumor grafts. HCI-002 tumor grafts from the experiment described in Figure 1C, 6 weeks after implantation, were fixed, sectioned, and stained with a PECAM-1/CD31 antibody. The relative endothelial cell counts and blood vessel numbers for sample HCI-002 were plotted. Bottom: Images of the blood vessel staining for each condition. Scale bar: 100 μm.

A.

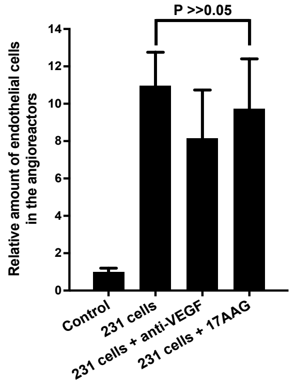

B.

Plates of cancer cells
(at least $2{\times}10^7$ cells required)

⬇

Conditioned Medium from cancer cells

⬇

~300g Centrifugations (3 × 5 mins) or 3.1 µm filtration
(clarify medium of cells and cell debris)

⬇

MVs are isolated from the medium using a Steriflip filter (0.1 or 0.22 µm poresize)

C. .

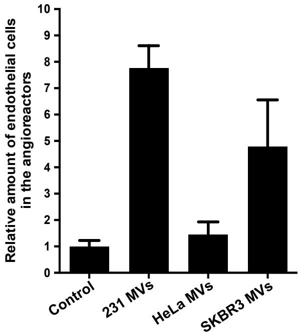

Amounts of endothelial cells in the angioreactors

Samples	Control	231 MVs	HeLa MVs	SKBR3 MVs
Avg. IF counts	1785 ± 399.7	13846 ± 1521	2587 ± 863	8541± 3164
Relative amounts	1.000 ± 0.224	7.757 ± 0.852	1.449 ± 0.483	4.785 ± 1.773

D. .

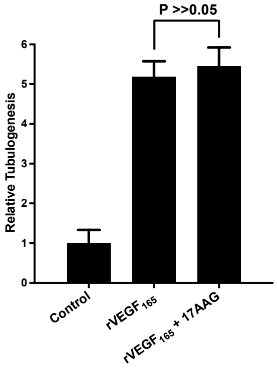

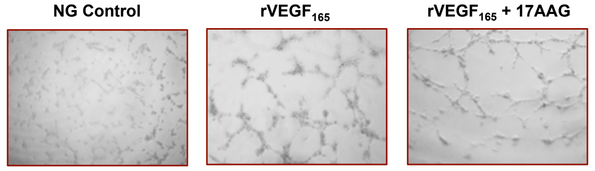

Relative stimulation of Tubulogenesis

Samples	NG Control	$rVEGF_{165}$	$rVEGF_{165}$ + 17AAG
Relative fold	1.000 ± 0.333	5.19 ± 0.385	5.45 ± 0.472

Supplementary Figure 2. A, The relative amounts of endothelial cells that entered implanted angioreactors that lacked any activators (vehicle control; histogram 1), or were loaded with MDAMB231 cells (5×10^4 cells/angioreactor) without (histogram 2) or with a pan inactivating VEGF antibody (200 ng/angioreactor; histogram 3) or with 10 mM 17AAG (histogram 4). **B,** Procedure for isolating MVs from conditioned medium (typically 2×10^7 MDAMB231 cells yielded ~5 mg of MV protein). Also, see 'Methods'. **C,** Left: Relative amounts of endothelial cells that entered angioreactors that lacked MVs (control; histogram 1), or contained MVs (2 mg total protein per angioreactor) from either MDAMB231 cells (histogram 2), HeLa cells (histogram 3), or SKBR3 cells (histogram 4). Right: Quantification of the number of endothelial cells that entered the angioreactor for the different conditions. **D,** Left: Relative amounts of tubulogenesis for HUVECs that were untreated (control; histogram 1) or treated with rVEGF (15 ng/ml) without (histogram 2) or with 10 mM 17AAG (histogram 3). Right: Images and quantification of the tubulogenesis assays.

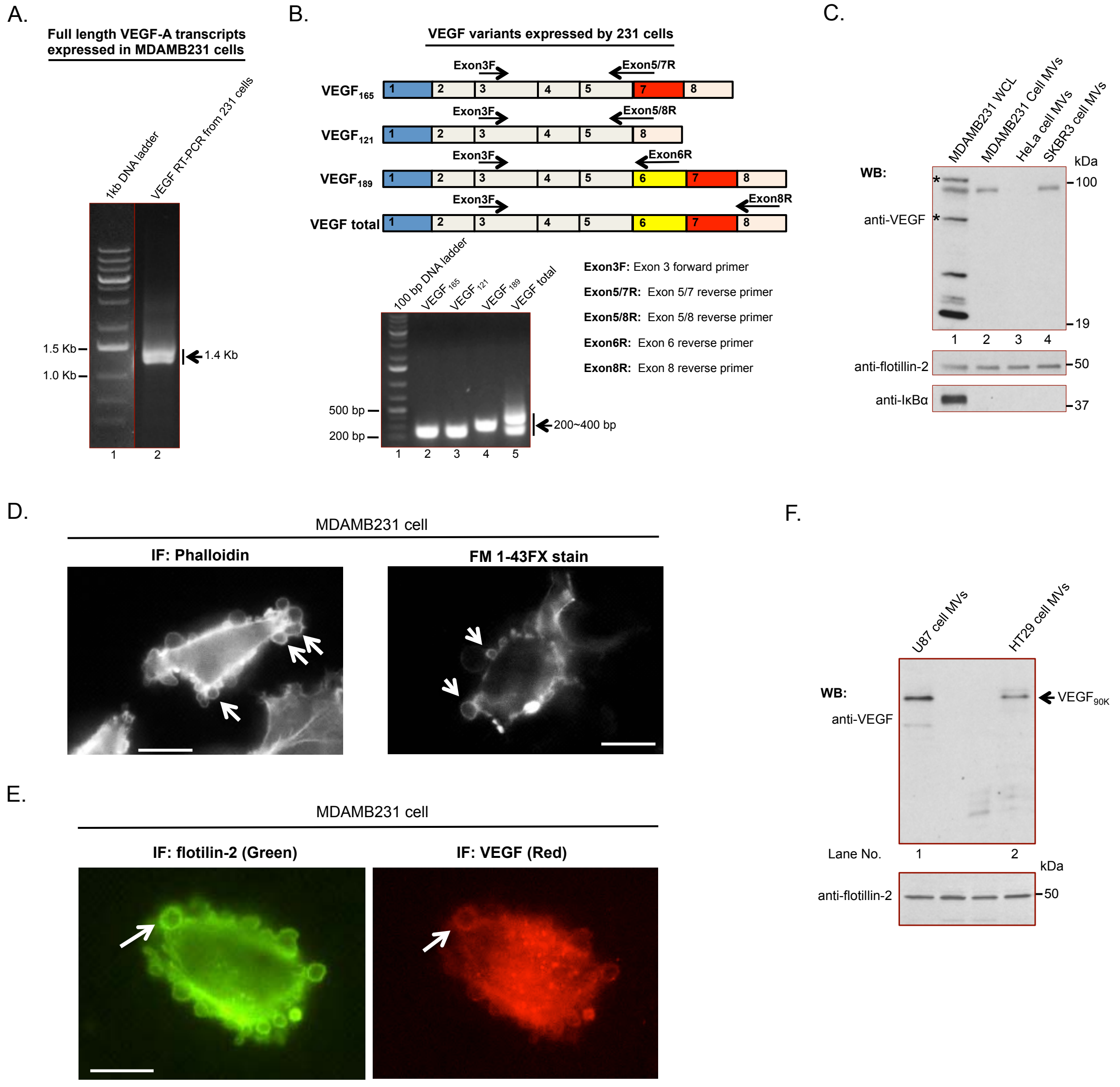

Supplementary Figure 3. **A,** The full length VEGF-A transcript including its 3'- and 5'-untranslated regions (UTRs) was amplified from MDAMB231 cells by RT-PCR. Sequencing of the resulting ~1.4 Kb PCR product (lane 2) showed that it matched the NCBI documented VEGF-A sequence. **B,** Top: The primer sets used to specifically amplify the indicated VEGF-A splice variants. Bottom: The resulting PCR products obtained (~200-400 bp; lanes 2-5). **C,** MDAMB231 whole cell lysates (WCL, lane 1) or MVs shed by MDAMB231 (lane 2), HeLa (lane 3), or SKBR3 cells (lane 4) were isolated and lysed. The MDAMB231 WCL show higher molecular mass forms of VEGF (indicated by asterisks) in addition to the 90 kDa $VEGF_{90K}$ species, as detected using a pan antibody recognizing VEGF. The MV lysates were immunoblotted with a pan antibody recognizing VEGF, and with antibodies against flotillin-2, and the cytosolic-specific marker IkBa. **D,** MDAMB231 cells were permeabilized and analyzed by immunofluorescent (IF) microscopy using Rhodamine-conjugated phalloidin (left panel) or the membrane dye FM 1-43FX (right panel) to detect MVs. Arrows indicate MVs. Scale bar: 5 μm. **E,** MDAMB231 cells were permeabilized and analyzed by immunofluorescent (IF) microscopy using antibodies against flotillin-2 and VEGF. Arrows indicate MVs. Scale bar: 5 μm. **F,** MVs shed from U87 (lane 1) or HT29 (lane 2) cells were isolated and lysed. The MV lysates (10 mg/sample) were immunoblotted with antibodies against VEGF and flotillin-2.

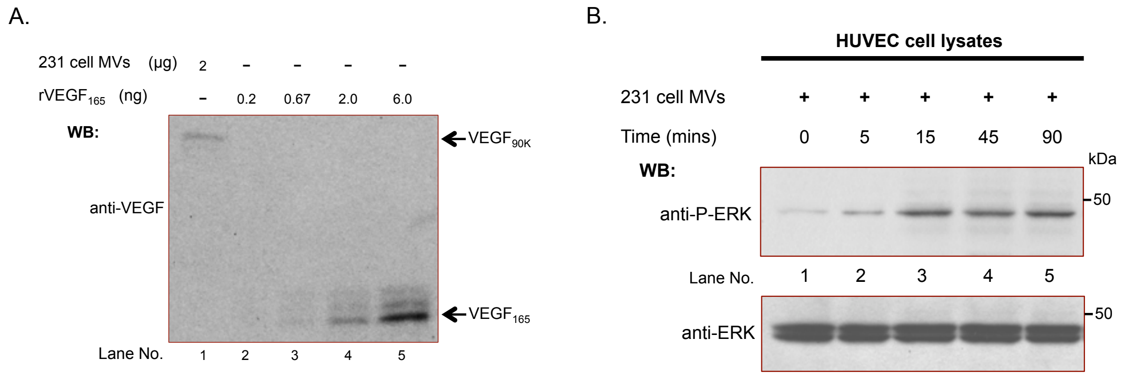

Supplementary Figure 4. **A,** Quantification of MV-associated $VEGF_{90K}$. MVs from MDAMB231 cells (2 mg of total protein, lane 1), or increasing amounts of rVEGF ($VEGF_{165}$, lanes 2-5), were immunoblotted with a pan VEGF antibody. Based upon the results from three independent experiments, we estimate that MVs contain ~1 ng of $VEGF_{90K}$ per 1 mg of total protein. **B,** Serum-deprived HUVECs were lysed after being exposed to MVs (5 mg/ml total protein) from MDAMB231 cells for the indicated periods of time (lanes 1-5). The lysates were then immunoblotted with antibodies that recognize phosphorylated (activated) and total ERK.

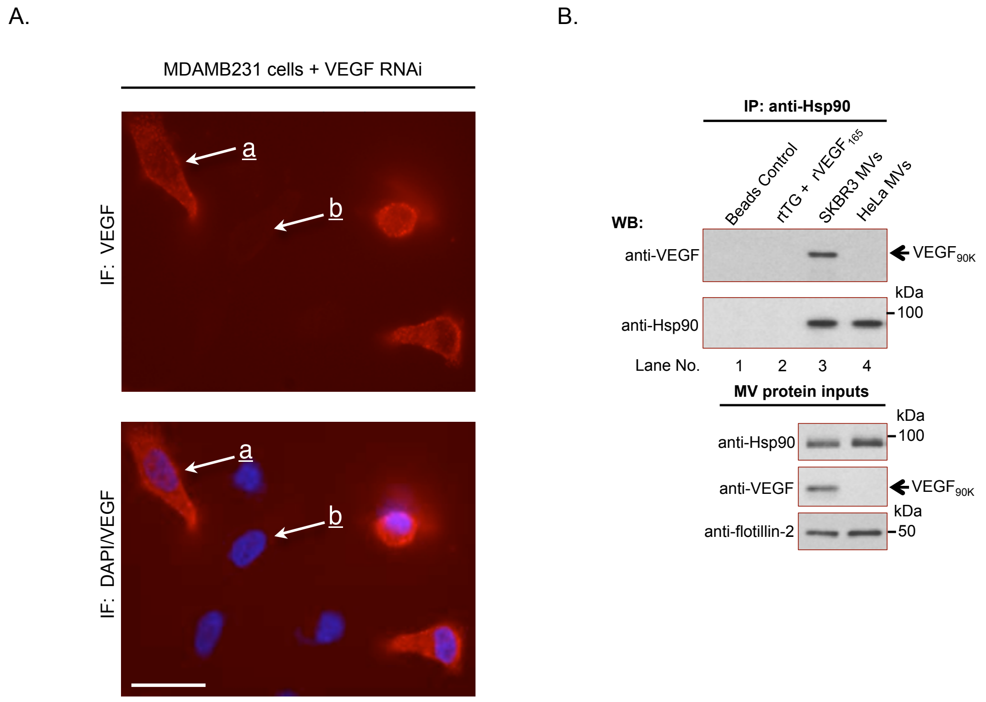

Supplementary Figure 5. **A,** MDAMB231 cells transfected with siRNAs targeting VEGF that yielded a partial knock-down of VEGF expression were analyzed by immunofluorescent (IF) microscopy using antibodies against VEGF and DAPI (to label nuclei) to validate the specificity of the VEGF antibody. Cells in which VEGF expression was knocked down were not labeled by VEGF antibody (compare cells expressing VEGF (arrows "a") with cells where VEGF expression was not detected due to the knock-down (arrows "b")). Scale bar: 20 μm. **B,** rVEGF165 (30 ng) was incubated with recombinant tTG (100 ng, lane 2) under optimal protein crosslinking conditions to generate VEGF$_{90K}$. MVs shed from serum-starved SKBR3 (lane 3) or HeLa (lane 4) cells were isolated and lysed. Immunoprecipitations (IPs) using an Hsp90 antibody (lanes 3 and 4) were performed on these samples, followed by Western blotting with antibodies against VEGF and Hsp90. Lanes 1 and 2 represented negative controls. The MV lysate inputs were Western blotted with antibodies against VEGF, Hsp90, and flotillin-2.

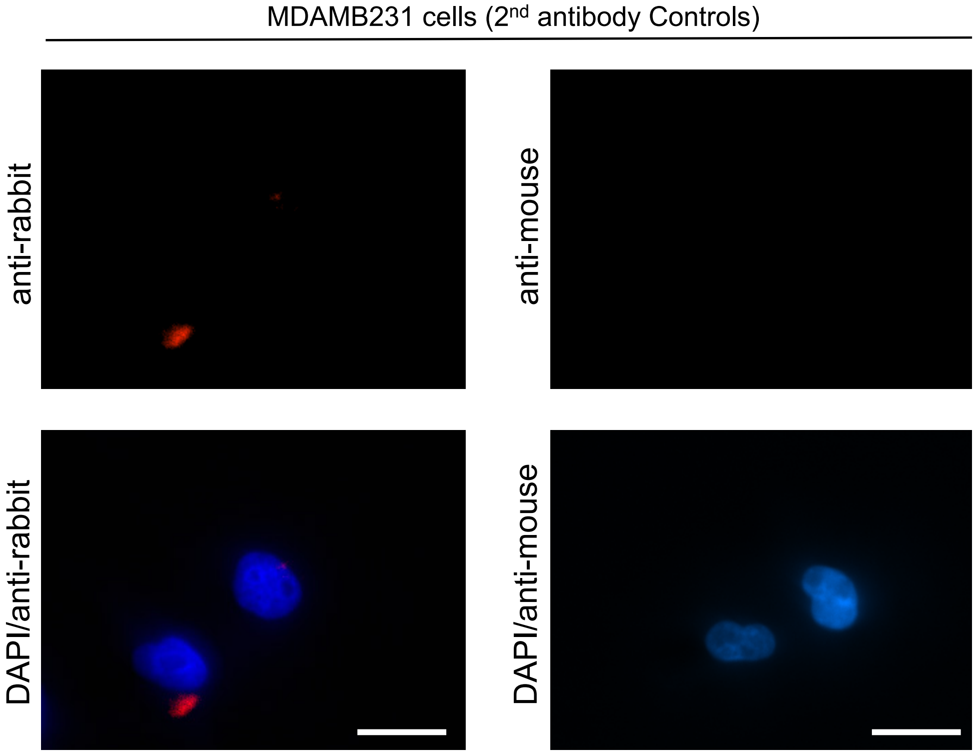

Supplementary Figure 6. IF images of MDAMB231 cells incubated with only IF-conjugated secondary antibody. Top left panel: Anti-rabbit secondary antibody control (the primary VEGF antibody was anti-rabbit). Top right panel: Anti-mouse secondary antibody control (the primary Hsp90 antibody was anti-mouse). DAPI was used to label nuclei (bottom panels). Scale bar: 10 μm.

A.

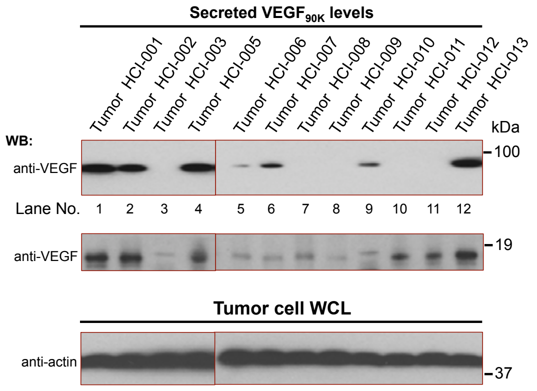

B.

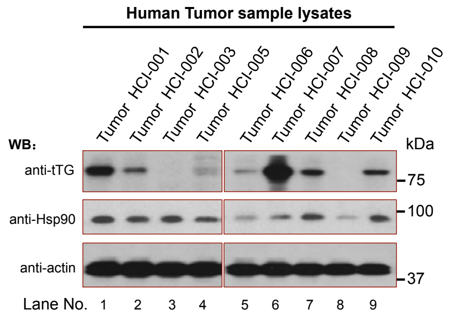

Supplementary Figure 7. A, Concentrated conditioned medium containing MVs from serum-starved cell cultures of PDX samples (described in Table 1) were immunoblotted using a pan VEGF antibody to detect $VEGF_{90K}$ (top panel) and a smaller VEGF species. Tumor cell lysates (WCL) were probed with an actin antibody to confirm the conditioned medium was from equivalent amounts of tumor cells (bottom panel). **B,** Cell lysates from PDX samples HCI-001 to HCI-003, and HCI-005 to HCI-010 were probed with antibodies against tTG, and Hsp90.

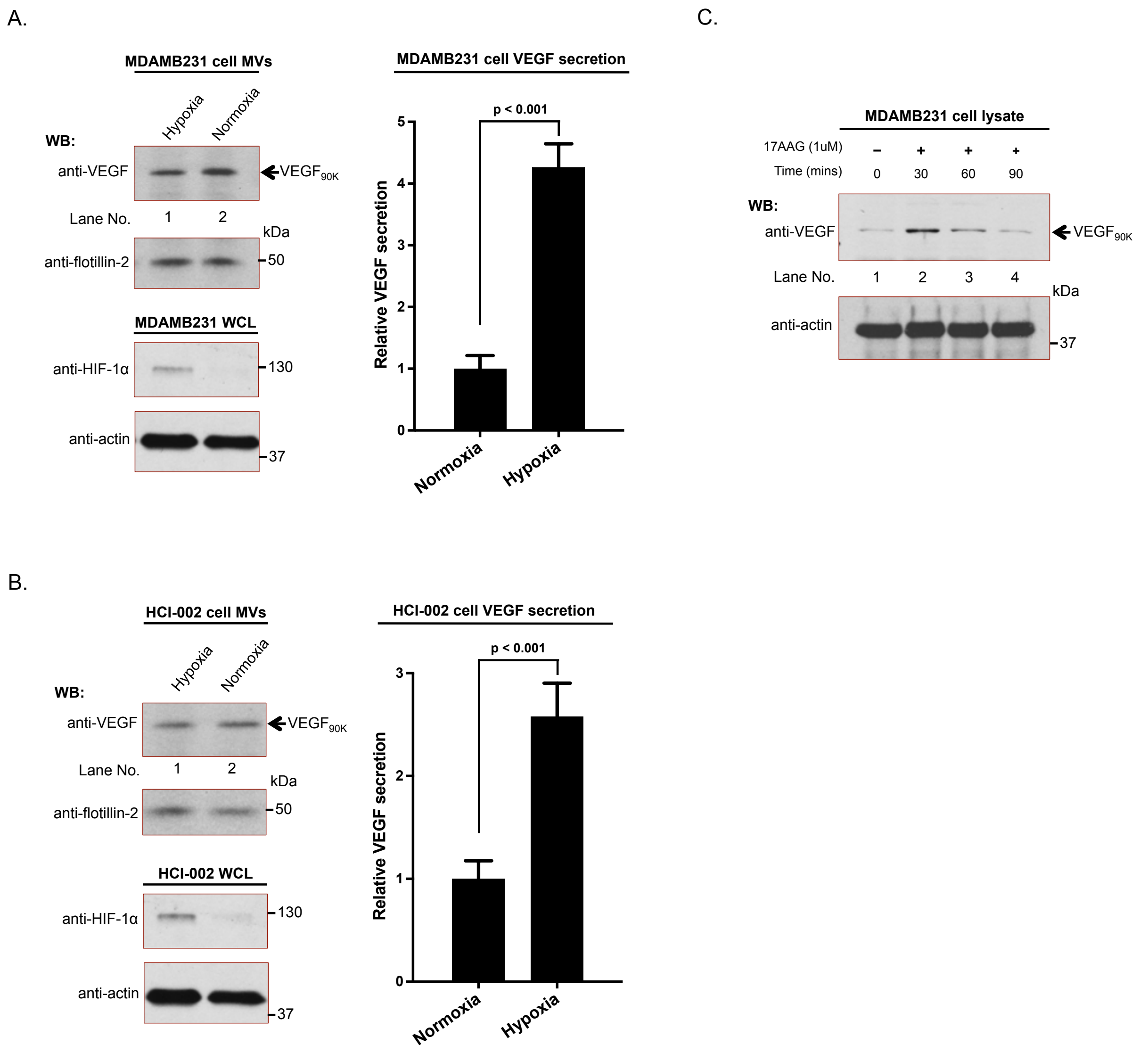

Supplementary Figure 8. A, MDAMB231 cells were cultured in CoCl2 (100 mM) containing medium (hypoxia) or medium without CoCl2 (normoxia). Left: MVs prepared from the same amount of MDAMB231 cells, treated with or without CoCl2, were immunoblotted with antibodies against VEGF and the MV marker flotillin-2 (top two panels). Lysates of the MDAMB231 cells were immunoblotted with antibodies against HIF-1a and actin (bottom two panels). Right: Plots of the relative VEGF secretion from the MDAMB231 cells, treated with or without CoCl2, were measured by VEGF ELISA. **B,** HCI-002 primary tumor cells were cultured in CoCl2 (100 mM) containing medium (hypoxia) or medium without CoCl2 (normoxia). Left: MVs prepared from the same amount of HCI-002 primary tumor cells, treated with or without CoCl2, were immunoblotted with antibodies against VEGF and the MV marker flotillin-2 (top two panels). Lysates of the HCI-002 primary tumor cells were immunoblotted with antibodies against HIF-1a and actin (bottom two panels). Right: Plots of the relative VEGF secretion from the HCI-002 primary tumor cells were measured by VEGF ELISA. **C,** MDAMB231 cells were lysed after being exposed to 17AAG for the indicated periods of time (lanes 1-4). The lysates were then immunoblotted with antibodies that recognize VEGF or actin.

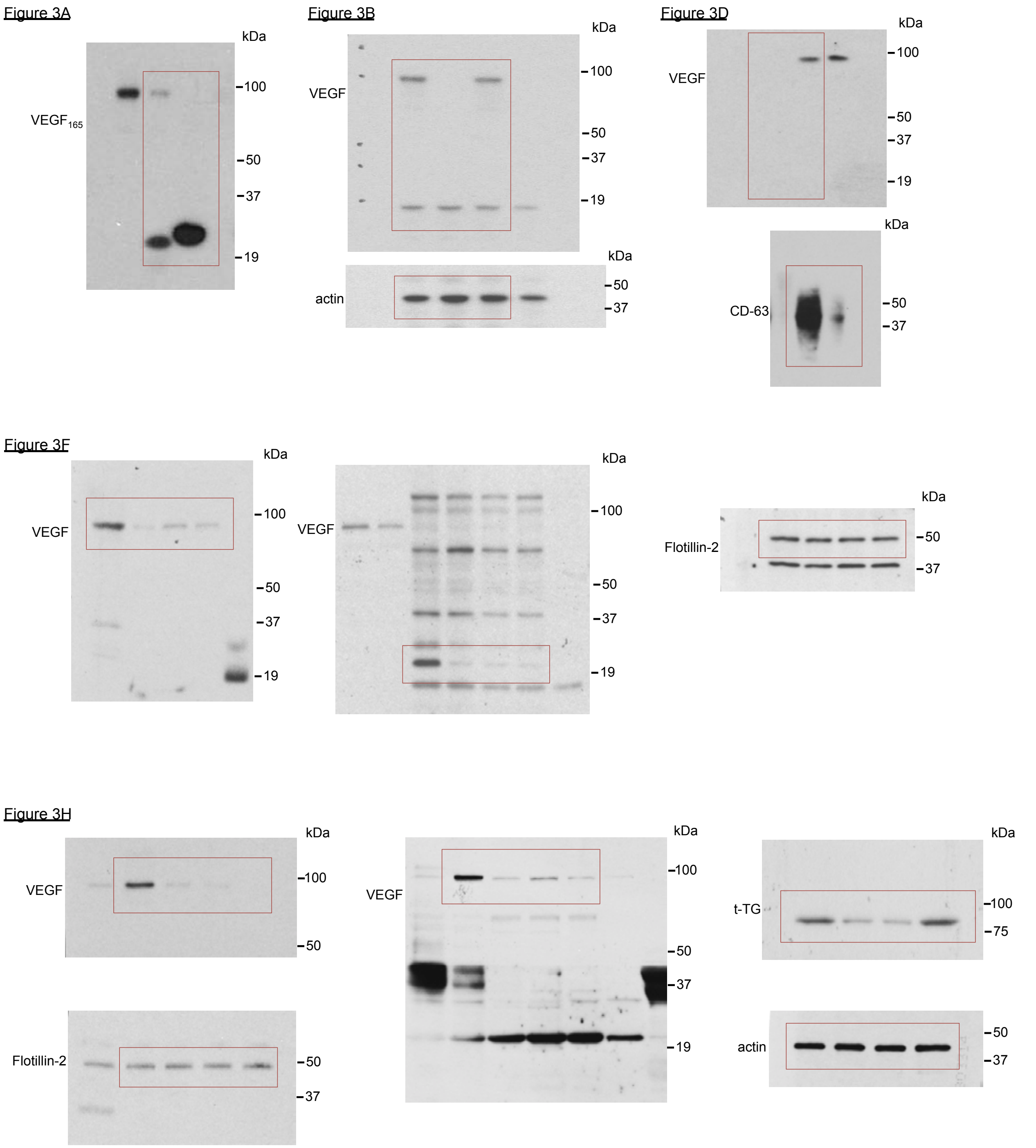

Supplementary Figure 9. Full, uncropped images of all immunoblots shown in the main figures. Molecular weight markers are indicated on the right of all blots. The red sections indicate blot results shown in the indicated figures.

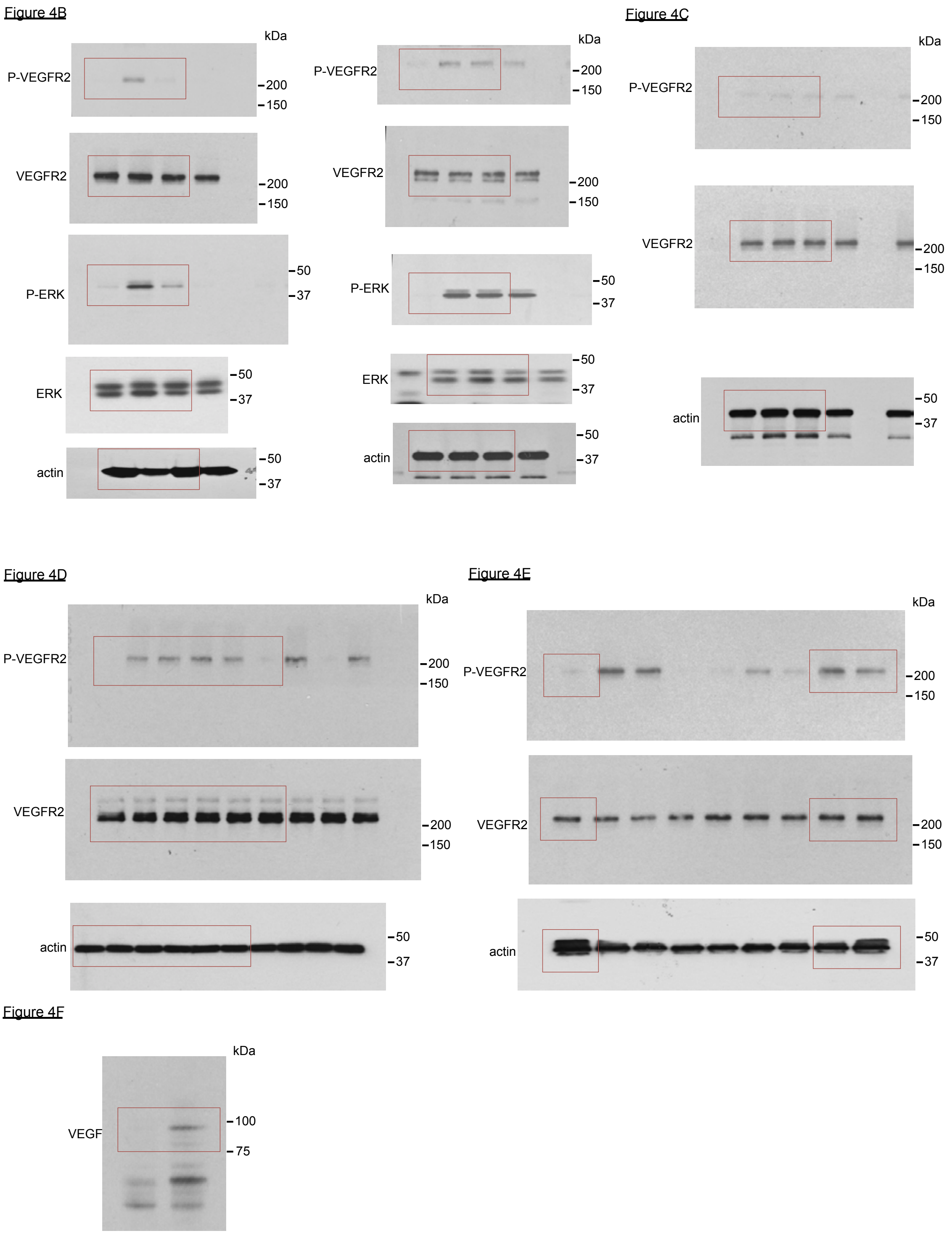

Supplementary Figure 9. Full, uncropped images of all immunoblots shown in the main figures. Molecular weight markers are indicated on the right of all blots. The red sections indicate blot results shown in the indicated figures.

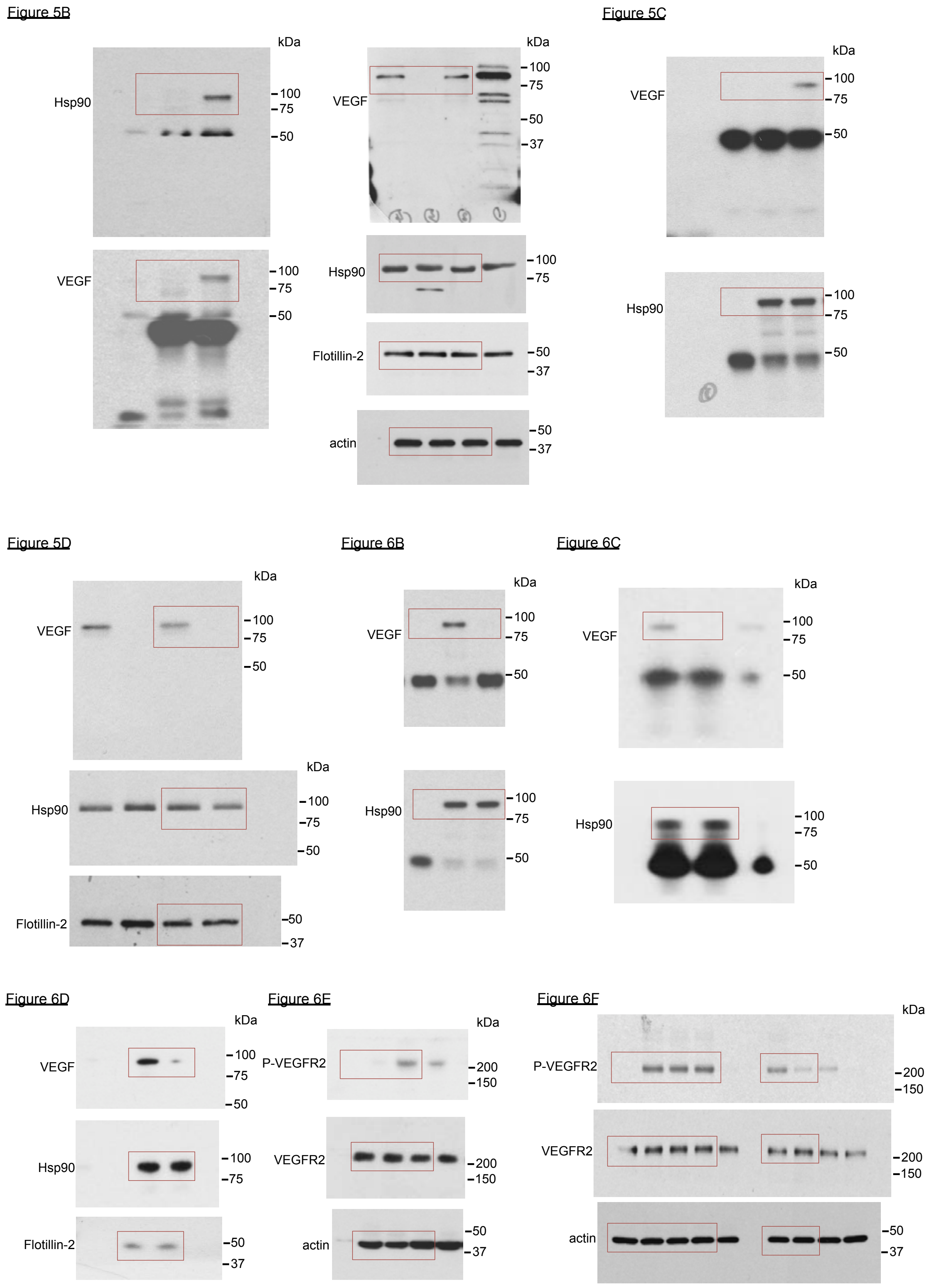

Supplementary Figure 9. Full, uncropped images of all immunoblots shown in the main figures. Molecular weight markers are indicated on the right of all blots. The red sections indicate blot results shown in the indicated figures.

Figure 6G

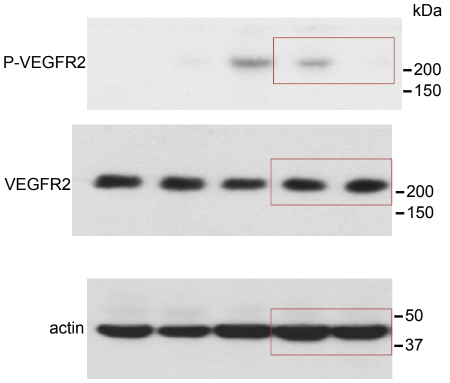

Figure 7A

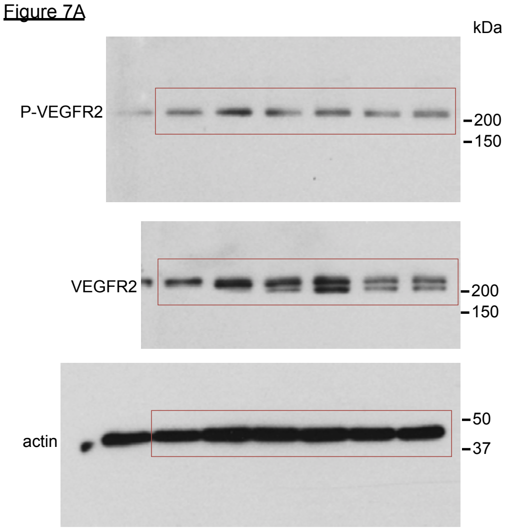

Figure 7B

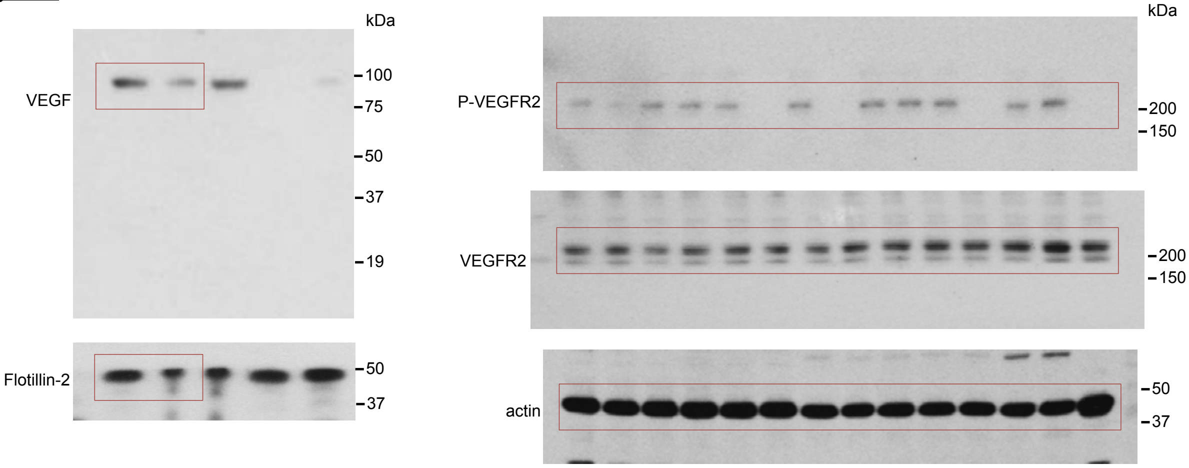

Figure 7C

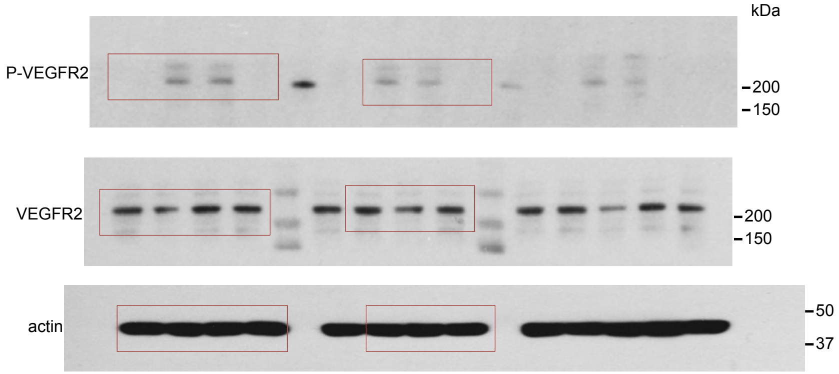

Supplementary Figure 9. Full, uncropped images of all immunoblots shown in the main figures. Molecular weight markers are indicated on the right of all blots. The red sections indicate blot results shown in the indicated figures.

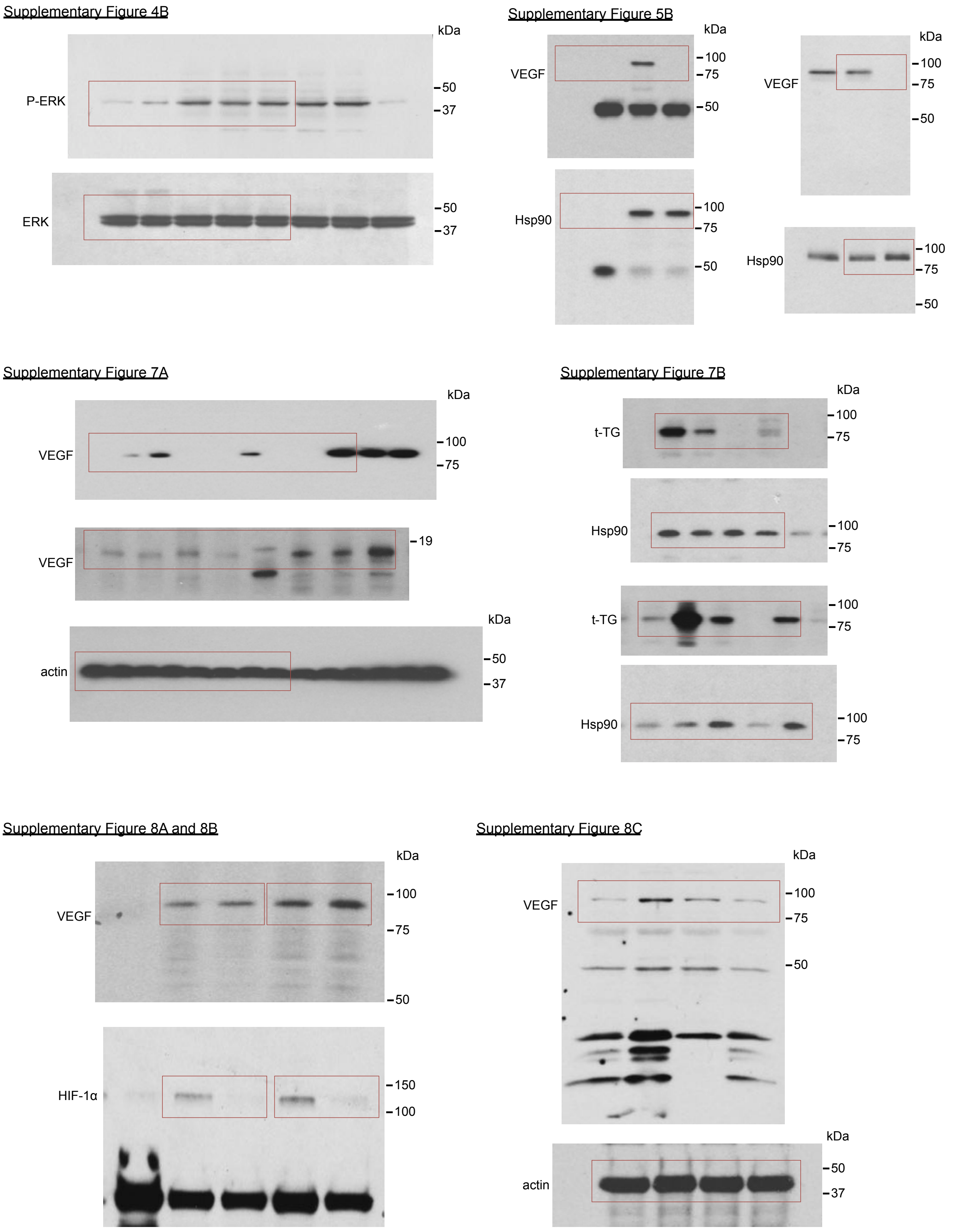

Supplementary Figure 9. Full, uncropped images of all immunoblots shown in the supplementary figures. Molecular weight markers are indicated on the right of all blots. The red sections indicate blot results shown in the indicated figures.
